# Global Perspective on Kidney Transplantation in Egypt

**DOI:** 10.34067/KID.0000000891

**Published:** 2025-06-11

**Authors:** Youssef M.K. Farag, Enass Elsayed

**Affiliations:** 1 Department of Epidemiology, Johns Hopkins Bloomberg School of Public Health, Baltimore, Maryland; 2 AstraZeneca Rare Disease Unit, Alexion, Boston, Massachusetts; 3 Harvard Medical School, Boston, Massachusetts; 4 Brigham and Women's Hospital, Boston, Massachusetts

**Keywords:** cadaver organ transplantation, CKD, kidney transplantation, organ transplant, pediatric kidney transplantation, renal transplantation, transplant outcomes, transplantation

## Introduction

According to the 2017 report by the Global Burden of Disease CKD Collaboration, the age-standardized prevalence of CKD in Egypt was 106 patients per 1000 population, and those patients were liable to develop kidney failure if they were not treated.

Individuals with kidney failure seek kidney transplantation (KT) as an alternative to regular hemodialysis, aiming to improve their quality of life. The first KT reported in Egypt was in 1976 at the Mansoura Urology and Nephrology Center. By 2008, the center performed 1967 KTs and exceeded 3000 KTs by 2019.^1^

From 1999 to 2010, there have been ongoing debates regarding the regulation of organ transplants in the Egyptian community, involving physicians, religious scholars, and influential thought leaders. In 2010, the Executive Regulation passed a law that allowed the transplant of organs or tissues from deceased individuals, but only between the Egyptians, if the deceased person had expressed a documented wish to donate before their death, and the procedure was expected to save the recipient's life.^2^ While Islamic religious leaders permitted the deceased donation, cultural and religious rejection among the community hindered the application of KT from deceased individuals in practice.^2,3^

Between 2011 and 2016, the annual new cases of KT in Egypt was around 1100, facilitated by 35 licensed centers on the basis of Egyptian regulations.^3^ Since then, the number of patients who underwent KT has increased, but the progress in the KT field remains relatively slow.^3^ Although 92% of Egyptian patients with kidney failure receiving hemodialysis were deemed suitable for transplantation, the 2020 Egyptian Renal Data System reveals only 0.3% of patients on hemodialysis received renal allografts within 1 year. This translates to a transplantation incidence rate of slightly more than three transplants per 1000 patient-years, compared with 36 per 1000 patient-years in the United States.^4^ The KT rate is affected by the waitlist of diverse health care sectors (*i.e*., private, university, government, and insurance), the ability of the recipient to afford the cost, and the availability of a suitable donor who fulfills the Egyptian legal requirements. The aim of this review was to shed light on the status, special considerations, and challenges of KT in Egypt.

## Donor Evaluation/Donation

Reflecting regional trends, the shortage of deceased donor programs in Egypt poses a significant challenge for organ transplants. According to the World Health Organization's donation and transplantation database, the percentage of KT from deceased donors in 2019 was 0.4% and 6.5% in Africa and the Middle East, respectively. This increases the risk of financially incentivized organ donation, resulting in social and economic repercussions because these donors often come from impoverished areas, representing a vulnerable demographic.^5^

It is common for Egyptian patients to decline kidney donations from close relatives because of concerns over potential health risks to the donor.^5^ However, a recent gender-focused analysis in Egypt revealed gender disparity in kidney donations, with six out of ten live kidney donations made by women.^6^ Contrary to the common assumption that familial donations are solely altruistic, various gender ideologies are accepted in Egypt, including scenarios of self-sacrificing mothers donating to their children, wives readily offering kidneys to husbands, and women making donation decisions to secure marriages and reproduction.^6^

## Clinical Outcomes

The 5- and 10-year survival rates after KT using a living donor at the Urology and Nephrology Center in Mansoura were 91% and 82%, respectively. Moreover, graft survival rates stand at 82% and 63%, respectively.^1^ Over a median follow-up of 8 years, death with a functioning graft was reported in 291 of 2952 (10%) patients, representing a major concern by causing 58% of total deaths, as the remaining 42% of deaths were associated with dialysis or retransplant.^7^ Death with a functioning graft was related mainly to cardiovascular events, infections, malignancies, and liver failure.^7^

In a retrospective study conducted at Cairo University on 282 patients who were followed up for at least 1 year, the incidence of early graft dysfunction was approximately 11%, with acute rejection accounting for 15.6% of patients.^8^ After the use of intravenous methylprednisolone for rejection treatment, a complete rejection reversal was noted in 29%, while partial reversal and renal impairment occurred in 49%, and graft failure resulted in hemodialysis in 23% of patients with rejection.^8^

## Post-Transplant Care

According to Cairo University's study, hypertension was the most common comorbidity after KT; however, hypertension was present among most patients before KT. Bone-related issues, such as osteoporosis, were identified in 5.7%, while 58% of patients reported different types of infectious diseases, with 32% of them caused by cytomegalovirus. In addition, 45% had nonspecific hepatic problems, such as mild elevation in liver enzymes, and 0.04% had liver failure.^8^ Reported malignancies included Kaposi's sarcoma, lymphoma, breast cancer, and bladder cancer.^8^ In addition, post-transplant diabetes mellitus was a common complication after KT, requiring a structured educational program. A majority of Egyptian KT recipients (98%) exhibited unsatisfactory or low measures of quality of life, influenced by various factors, *i.e*., age, marital status, education, employment status, and adverse effects of immunosuppression medications.^9^

## Special Situations

### Hepatitis C Virus

Hepatitis C virus (HCV) is a major health concern in Egypt, with the highest incidence reported globally. Chronic HCV may increase the risk of renal impairment and result in glomerular disease in native or transplanted kidneys. Furthermore, KT recipients are at a higher risk of chronic HCV, likely resulting from blood transfusions or their immunosuppressed state, potentially leading to graft loss and increasing mortality risk. By contrast, while HCV is not associated with patient and graft survival, it may be associated with long-term chronic rejection attributable to immunity modulation.

HCV seropositivity was observed in nearly 30% of the Egyptian patients on hemodialysis. Among those, 75% tested positive for polymerase chain reaction.^4^ On the basis of Cairo University's study, 16.6% of the recipients tested positive for anti-HCV antibodies with negative PCR for HCV. In addition, 1.4% of these recipients received an allograft from a donor who was anti-HCV positive, while the remaining donors were seronegative for HCV.^8^ The burden of HCV on KT has decreased due to newly available medications. Direct-acting antivirals were effective in treating infected KT recipients with genotype-4 HCV, the most common genotype in Egypt, with no adverse effects on graft survival or functionality.

### Fasting Ramadan

Ramadan fasting after 1 year from KT may be safe provided that the kidney recipients have good and stable baseline renal function within the level of CKD 3 or better. The Ramadan and Kidney Diseases Task Force recommends against fasting in the first year after KT. However, if kidney function remains stable and fasting does not disrupt the immunosuppressant medication schedule, then a KT recipient may fast under close medical supervision.

## Cost and Coverage

Health care financing in Egypt is facilitated through insurance, government funds, and, primarily, patient expenses. The government-covered expenses related to KT in Egypt include approximately $370 for operative evaluation and desensitization as needed, $1400 for the procedure, and $1440 for postoperative care. Private health care plays a crucial role in alleviating the strain on the Ministry of Health. However, donor preparation and operative stage may range from $500 to $8000 USD. Subsequent post-transplant care varies on the basis of diverse patient factors.

## Practice and Legislation for Kidney and Organ Donation

Traditionally, urologists and vascular surgeons perform KT, while nephrologists manage post-transplant recipient care. Typically, a hospital stay of 7 days is required after KT. The Higher Committee for Organ Transplant oversees all organ and tissue transplant procedures.

While regulations and oversight for KT were limited in Egypt before 2010, currently the law governing organ and tissue transplantation in Egypt allows live donations, restricting donations from Egyptians to foreigners, permitting exceptions for spouses in marriage lasting over 3 years. Nonrelated donations are allowed only when related donations involving second-degree kin are unfeasible. Donors must be younger than 60 years, mandating blood group and tissue matching for an anticipated success rate, with no financial compensation. Egypt has implemented laws criminalizing organ trafficking, subjecting all contributors to penalties, including involved medical professionals and facilities.^3^

## Challenges

While organ shortages are a major challenge worldwide, Egypt faces additional economic and cultural challenges. Religious beliefs significantly influence the Egyptian public's perspective on KT, posing substantial barriers to establishing a national transplantation program that aligns with these values. In addition to overseeing kidney failure reporting—on the basis of voluntary or semivoluntary input from hemodialysis units—the Egyptian Society of Nephrology and Transplantation leads the deceased donor guidelines, balancing perspectives from recipients, patients, donors, and governing bodies. While the Egyptian Society of Nephrology and Transplantation guidelines are thorough, campaigns with physicians, politicians, and influencers are crucial for advocating organ donation after death, with emphasis on religious and humanitarian perspectives.^2,10^

## Supplementary Material

SUPPLEMENTARY MATERIAL
