# Supplementary material for: Global Perspective on Kidney Transplantation in Egypt
Source: Kidney360. 2025 Jun 11;6(12):2251–3. doi: 10.34067/KID.0000000891 (PMC12708395; doi:10.34067/KID.0000000891)
Supplement: SUPPLEMENTARY MATERIAL [file kidney360-6-2251-s001.pdf]

ASN American Society of Nephrology | JASN Journal of the American Society of Nephrology | KIDNEY360 Accessing Our World From Every Angle | CJASN Clinical Journal of the American Society of Nephrology

ASN Journal Disclosure Form

As per ASN journal policy, I have disclosed any financial relationships or commitments I have held in the past 36 months as included below. I have listed my Current Employer below to indicate there is a relationship requiring disclosure. If no relationship exists, my Current Employer is not listed.

E. Elsayed has nothing to disclose.

I understand that the information above will be published within the journal article, if accepted, and that failure to comply and/or to accurately and completely report the potential financial conflicts of interest could lead to the following: 1) Prior to publication, article rejection, or 2) Post-publication, sanctions ranging from, but not limited to, issuing a correction, reporting the inaccurate information to the authors' institution, banning authors from submitting work to ASN journals for varying lengths of time, and/or retraction of the published work.

Name: Enass Sayed Elsayed
Manuscript ID: K360-2024-000535R2
Manuscript Title: "Global perspective on kidney transplantation in Egypt,"
Date of Completion: May 27, 2025
Disclosure Updated Date: May 27, 2025

ASN American Society of Nephrology | JASN Journal of the American Society of Nephrology | KIDNEY360 Accessing Our World From Every Angle | CJASN Clinical Journal of the American Society of Nephrology

ASN Journal Disclosure Form

As per ASN journal policy, I have disclosed any financial relationships or commitments I have held in the past 36 months as included below. I have listed my Current Employer below to indicate there is a relationship requiring disclosure. If no relationship exists, my Current Employer is not listed.

Y. Farag reports the following:
Employer: Alexion AstraZeneca Rare Disease Unit

I understand that the information above will be published within the journal article, if accepted, and that failure to comply and/or to accurately and completely report the potential financial conflicts of interest could lead to the following: 1) Prior to publication, article rejection, or 2) Post-publication, sanctions ranging from, but not limited to, issuing a correction, reporting the inaccurate information to the authors' institution, banning authors from submitting work to ASN journals for varying lengths of time, and/or retraction of the published work.

Name: Youssef MK Farag
Manuscript ID: K360-2024-000535R1
Manuscript Title: Global perspective on kidney transplantation in Egypt
Date of Completion: April 10, 2025
Disclosure Updated Date: May 21, 2024
